# ARR1 and AHP interactions in the multi-step phosphorelay system

**DOI:** 10.3389/fpls.2025.1537021

**Published:** 2025-02-27

**Authors:** Linh H. Tran, Milosz Ruszkowski

**Affiliations:** Institute of Bioorganic Chemistry, Polish Academy of Sciences, Poznan, Poland

**Keywords:** multi-step phosphorelay, response regulator, cytokinin signaling pathway, histidine-containing phosphotransfer protein, signaling cascade

## Abstract

Plants use multi-step phosphorelay (MSP) systems in response to exogenous and endogenous stimuli. Cytokinin and ethylene are among the factors that engage MSP signaling cascades but examples independent of phytohormones also exist. The MSP signaling involves four consecutive phosphorylation events at: (i) the kinase domain of the sensory histidine kinase, (ii) the receiver domain of the latter protein, (iii) the histidine-containing phosphotransfer protein, and (iv) the response regulator. In *Arabidopsis thaliana*, there are eight canonical histidine kinases, five histidine-containing phosphotransfer proteins (AHPs), one pseudo AHP, and 23 response regulators (ARRs). This redundancy suggests complex interactions between signaling pathways, including those involved in phytohormone cross-talk. To bring new insights at the molecular level, we investigated the structural and biophysical characteristics of the AHP1/ARR1 complex. ARR1, a type-B ARR, contains the GARP domain for DNA binding, in addition to the canonical receiver domain that mediates AHP1 interaction. We compared the ARR1 affinities across all five active AHPs and found a modest, two-fold higher affinity for AHP1. This result suggests that while ARR1 shows a slight preference for AHP1, it can also interact with AHP2-5, which potentially makes ARR1 a central node in signaling and a cross-talk modulator. In addition, we discuss the oligomerization state of AHP and related proteins utilizing all available experimental data to conclude that free AHPs are most likely monomeric.

## Introduction

**Figure 1:**
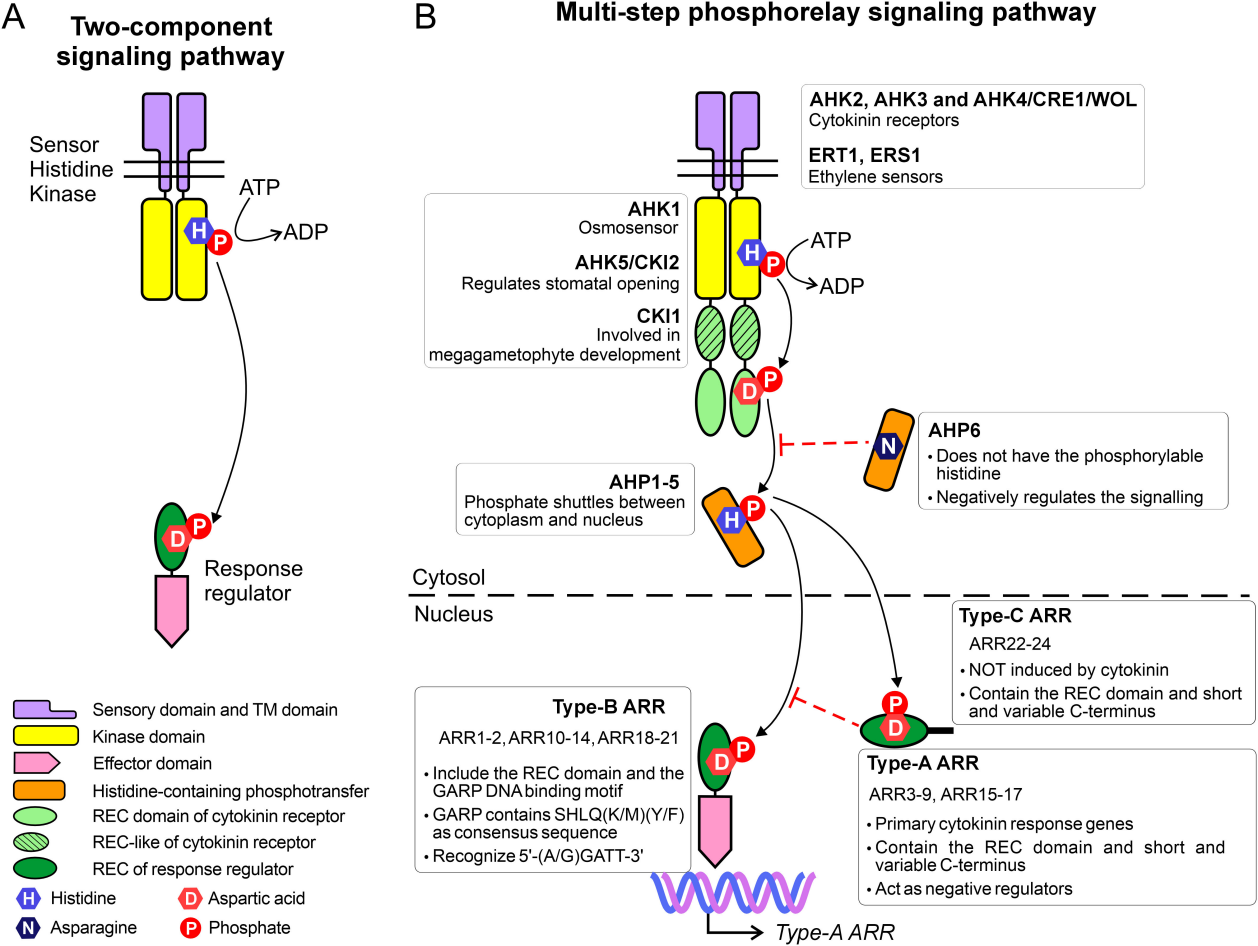
Comparison between **(A)** the two-component system and **(B)** the multi phosphorelay system.

In both prokaryotes and eukaryotes, response regulators (RRs) are essential components of signaling pathways, which makes them key players in the response to changing environmental conditions (Stock et al., 1990). In prokaryotes, several signaling pathways are governed by the so-called two-component system (TCS) that includes a sensory histidine kinase (HK) and a response regulator **Figure 1A** (Stock et al., 2000; Buschiazzo and Trajtenberg, 2019). While phosphorylation of Ser, Thr, or Tyr is more stable, TCS relies on the phosphorylation relay between His and Asp residues. Upon external stimuli, the kinase domain first undergoes auto-phosphorylation, subsequently transferring phosphate directly to the RR. This phosphorylation activates the RR, enabling it to initiate transcription of specific target genes. Even in the simplest setup, both HK and RR exhibit a modular architecture (Mizuno, 1997). The HK contains a sensory (input) domain that detects external stimuli, leading to autophosphorylation of a conserved histidine residue within the core kinase domain (Stock et al., 2000). The RR comprises a receiver domain (REC), which is phosphorylated by the kinase at a conserved aspartate residue, and an effector domain, which typically includes a DNA-binding motif.

The TCS has evolved into the multi-step phosphorelay system (MSP) in plants (**Figure 1B**), where it plays a central role in regulating plant growth, development, and stress responses (Grefen and Harter, 2004). The MSP also consists of HK and RR, and in addition, the histidine-containing phosphortranfer proteins, which act as the shuttle between HK and RR. In *A. thaliana*, there are eight canonical histidine kinases, including AHK1-5, CKI1, ETR1 and ERS1. Among them, AHK2, AHK3, and AHK4 (also known as CRE1/WOL) function as cytokinin receptors, mediating cytokinin signaling. CRE1/AHK4 is the first cytokinin receptor identified (Inoue et al., 2001; Ueguchi et al., 2001b; Yamada et al., 2001) and it is expressed mainly in roots (Mahonen et al., 2000). Meanwhile, *AHK2* and *AHK3* can be found in other organs at varying levels of transcripts (Ueguchi et al., 2001a). The class-I ethylene receptors, ETR1 and ERS1, also belong to the (canonical) HK family, whereas the class-II ethylene receptors are considered AHK-like proteins (O’malley et al., 2005). AHK1 serves as an osmosensor, detecting the water stress not only in early vegetative stages but also at the seed formation stage (Wohlbach et al., 2008). CKI1, which was previously thought to participate in cytokinin signaling, is now recognized for its role in megagametophyte development (Hejatko et al., 2003). Lastly, AHK5 (also known as CKI2) plays a key role in regulating stomatal opening, contributing to plant responses to environmental cues (Desikan et al., 2008).

The second element in the MSP system, AHP, receives a signal (phosphate) from the receiver (REC) domain of HK and translocates into the nucleus to phosphorylate the REC domain of the RR. There are five active AHPs (AHP1-5) which posses a conserved histidine residue and work as phosphate shuttles between the cytoplasm and nucleus (Suzuki et al., 1998, 2000; Hwang and Sheen, 2001). AHP6 lacks the phosphorylatable histidine and acts as the inhibitor of cytokinin signaling (Mahonen et al., 2006). AHP1 is expressed mainly in roots, AHP2,3 exist in all organs, AHP5 has been found in roots and leaves, whereas AHP4 has been difficult to detect (Suzuki et al., 1998; Tanaka et al., 2004).

The third element, plant RRs, exhibit remarkable diversity. For instance, there are 23 RRs and nine pseudo-RRs in *A. thaliana*, whose RRs are named ARRs. All ARRs are localized in the nucleus (Imamura et al., 2001; Hosoda et al., 2002), with the exception of ARR3, ARR16 (Dortay et al., 2008), and ARR22 (Horak et al., 2008), which are also present in the cytosol. ARRs are classified into types A, B, and C based on sequence similarity, and domain architecture. Interestingly, ethylene, can induce type-A ARRs to repress cytokinin signaling (Shi et al., 2012). Type A-ARRs (10 members) contain solely the REC domain and a short but variable C-terminus. The C-terminal fragment contains a nuclear localization signal; truncation of this region prevents the protein from entering the nucleus but does not affect the phosphorylation capability (Imamura et al., 2001). As primary cytokinin response genes, all type-A ARRs respond quickly to exogenous cytokinins within hours (Kiba et al., 1999; D’agostino et al., 2000).

Type-C ARRs derive from type-A ARRs. The only two members, ARR22 and ARR24, also contain only the REC domain. However, based on sequence similarity, type-C ARRs are closely related to REC in histidine kinases (Kiba et al., 2004; Schaller et al., 2008).

Type-B ARRs contain the so-called GARP (Golden 2, ARR-B, Psr1) DNA-binding motif, in addition to the N-terminal REC domain. At the first glance, the GARP motif resembles the MYB superfamily. However, a more detailed analysis revealed that they are distant relatives (Safi et al., 2017). More precisely, the MYB members possess three repeats of DNA-binding motifs, each containing a hallmark of three Trp residues separated by 18-19 residues. Meanwhile, the GARP motif has only one repeat and a single Trp. Moreover, GARP contains the SHLQ(K/M)(Y/F) consensus sequence, which slightly differs from the characteristic motif of MYB-related proteins (SHAQK(Y/F)F) (Safi et al., 2017). Type-B ARRs also vary in their preference for DNA motifs. For instance, ARR1 and ARR10 preferentially bind to 5’-AGATT-3’ (Sakai et al., 2000; Hosoda et al., 2002) while ARR11 favors 5’-GGATT-3’ (Imamura et al., 2003).

Based on their expression pattern, type B-ARRs are further divided into three subclasses: BI, BII, and BIII (Mason et al., 2004). Subclass BI includes ARR1-2, ARR10-12, ARR14, and ARR18, which are expressed in all the plant tissues. The authors noted that ARR1 is highly expressed in roots. In contrast, the ARRs of subgroups BII (ARR13 and ARR21) and BIII (ARR19 and ARR20) are expressed mainly in the reproductive organs (Mason et al., 2004). Nonetheless, the universal feature of type-B ARRs is that they serve as positive regulators and can induce expression of response genes. Interestingly, type-B ARRs often regulate the expression of type-A ARR genes by binding to their promoter regions, thus establishing the negative feedback loop in the signaling pathways (Taniguchi et al., 2007).

In the MSP cascade, AHPs directly interact with ARRs, including AHP1 that has been shown to interact with ARR1 using two-yeast hybrid assay (Dortay et al., 2006). Some of the AHP examples have been shown to phosphorylate ARRs; for instance AHP2 and AHP5 can phosphorylate ARR11 and ARR22, respectively (Imamura et al., 2003; Kiba et al., 2004). However, the structure of a complex for any AHP/ARR pair (or HPt/RR for other plant species) has remained unknown, making it difficult to understand the molecular determinants of the binding and/or predict the selectivity or promiscuity of such interactions. Until recently, the only element of an ARR protein structure solved experimentally was the NMR structure of the GARP motif (Hosoda et al., 2002). Examining the predictions made by AlphaFold (Jumper et al., 2021) partially explains the difficulties because almost every type-B ARR contains a long unstructured loop that might only fold upon binding to its partners. In 2024, the structure of the REC domain and GARP motif of ARR1 has been published by Zhou et al., who revealed the molecular details of DNA recognition (Zhou et al., 2024). Our work focused on the upstream complex between ARR1 and AHP1, both of which are key representatives of the respective elements in the MSP systems. It is also important to note that, based on expression patterns, AHP1 and ARR1 are co-expressed in all plant tissues, including roots, where they colocalize with AHK4. This shared localization supports the relevance of AHP1/ARR1 interaction within cytokinin signaling. However, their involvement is not limited to this pathway, as both proteins have been reported to participate in cytokinin-independent signaling cascades (Urao et al., 2000; Mira-Rodado et al., 2012).

## Results and discussion

### Overall properties of the AHP1/ARR1-REC-GARP complex structure

Our construct for ARR1-REC-GARP (ARR1-RG) spans residues 38-296 in the ARR1 sequence (Uniprot ID: Q940D0), whereas AHP1 was produced as the full-length protein (Uniprot ID: Q9ZNV9). The complex was obtained by mixing the two components, followed by purification using size-exclusion chromatography (SEC). The two proteins eluted together on SEC, with a shorter retention volume compared to either one (not shown). The AHP1/ARR1-RG complex crystallized in the *P*2_1_2_1_2_1_ space group with the unit cell dimensions of 84.5 × 132.6 × 160.7 Å. The crystals were very fragile, owing to 74% of water content based on the Matthews coefficient calculation; tens of crystals were tested before reaching satisfactory diffraction properties. From the best crystal, the resolution was truncated anisotropically to 2.9 Å; the data collection and refinement details are shown in **Table 1**. The asymmetric unit (ASU) includes two complexes, each consisting of one AHP1 molecule and one ARR1-RG. While the electron density map clearly defines the entire AHP1 proteins, the region spanning 77 residues (from 159 to 235) in ARR1-RG is not visible, indicating the flexibility of the fragment that links the REC domain and the GARP motif. The ASU also contains two oxamic acid molecules (from the crystallization solution), three ethylene glycol molecules, and two glycerol molecules. Only seven water molecules were placed in the structure owing to the limited data resolution.

**Table 1:** Diffraction data and refinement statistics.

Data collection	AHP1/ARR1-REC-GARP		
Beamline	P13 beamline of the PETRA III storage ring at DESY Hamburg		
Wavelength (Å)	1.0000		
Temperature (K)	100		
Space group	*P*2_1_2_1_2_1_		
Unit cell parameters a/b/c (Å)	84.5/132.6/160.7		
	Overall^a^	Inner^a^	Outer^a^
Resolution (Å)	80.34-2.87	80.34-8.97	3.15-2.87
Unique reflections	30328	1513	1517
Multiplicity	13.6	11.5	14.1
Ellipsoidal completeness (%)	94.6	99.9	68.6
Spherical completeness (%)	72.4	99.9	15.3
R_merge_	0.11	0.07	0.92
<I/σ(I)>	15.5	31.0	3.2
CC(1/2)	0.998	0.997	0.810
Refinement			
R_free_ reflections	1031		
No. of non-H atoms			
protein	5390		
solvent (water/other)	45		
R_work_/R_free_ (%)	19.13/22.86		
RMSD from ideal geometry			
bond lengths (Å)	0.010		
bond angles (°)	1.149		
Ramachandran statistics (%)			
favored/allowed/outliers	98.48/1.52/0.0		
PDB ID	9H6E		

a Data processing statistics are given separately for: all reflections (left column), inner shell (middle column), and outer shell (right column).

### The ARR1-REC and GARP structure and interface

**Figure 2:**
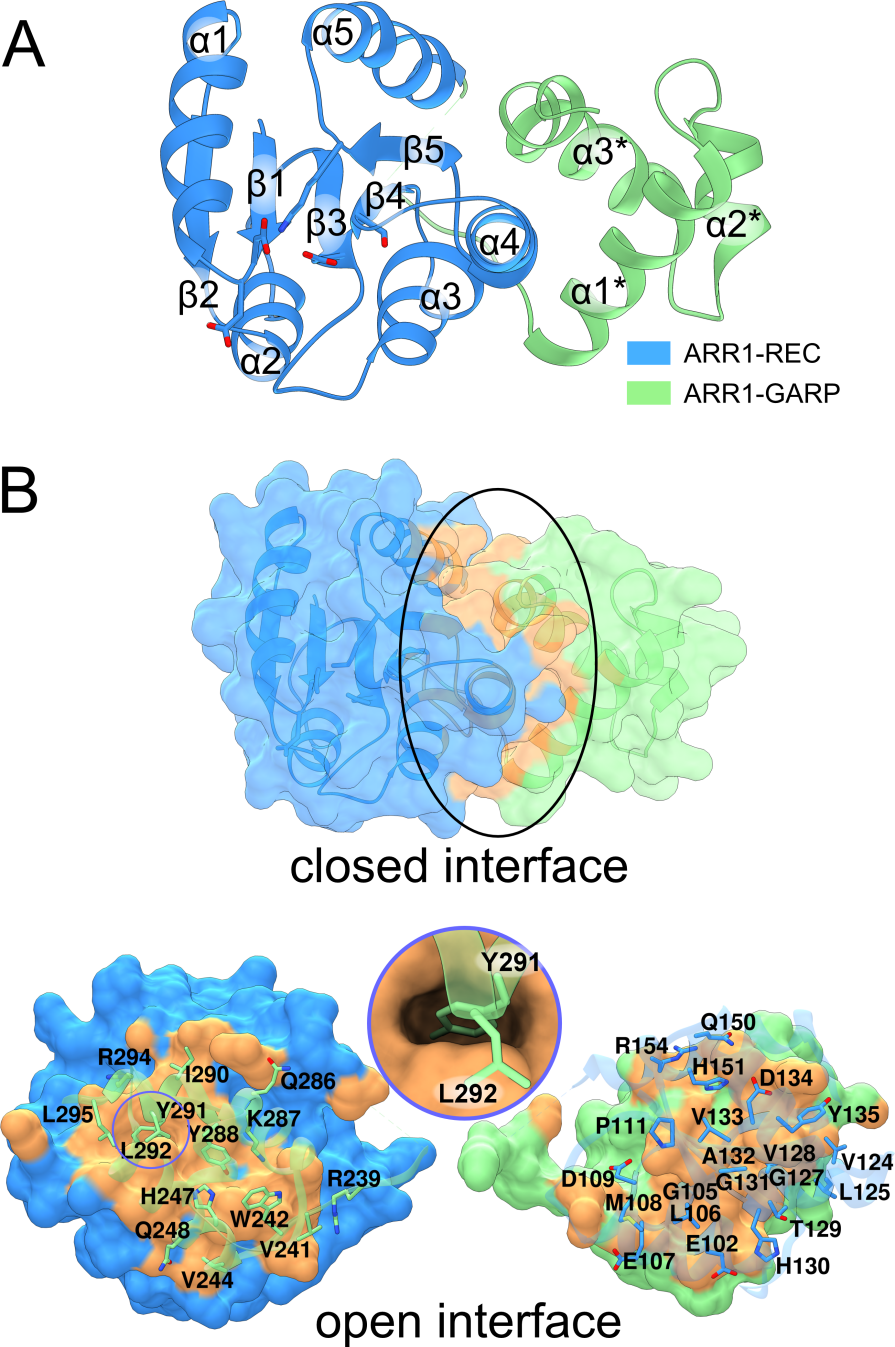
The structure of ARR1-REC-GARP. In **(A)**, the structure is presented in cartoon representation, with the receiver domain in blue and the GARP-DNA binding motif in light green. **(B)** shows the extensive interface (orange surface, marked by black ellipse) between REC and GARP. The model has been artificially broken apart (bottom) to visualize the residues involved in the interface. The circle inset illustrates Tyr291 reaching deep into the ARR1-REC domain.

ARR1-RG contains the REC domain (residues 38-158) and the GARP motif (residues 236-296). The REC domain has five β/α motifs and adopts the typical fold as in the bacterial REC family (Gao et al., 2019), where an anti-parallel β-sheet (β2-β1-β3-β4-β5) is planked between two layers of α-helices (α1-α5 and α2-α3-α4) (**Figure 2A**). The GARP has the canonical helix-turn-helix motif of three α-helices which are held together by a hydrophobic core made out of Phe250, Val254, Ile267, Met271, Val281, and Leu285. The lengths of the loops are approximately five residues, allowing a certain degree of flexibility and/or adjustments required for efficient binding. Notably, the α3 helix is slightly bent compared with that in the published GARP structure of ARR10 (Hosoda et al., 2002). Despite being separated by 77 residues (159-235) in the ARR1 sequence, the REC domain and GARP motif interact with one another through a vast interface of 1044 Å^2^ [calculated by PDBePISA (Krissinel, 2010)], as shown in **Figure 2B**. This interface involves up to fourteen hydrogen bonds and seven salt bridges (depending on the subunit in our crystal structure). The Tyr291 residue from the GARP motif reaches deep into a hydrophobic pocket of the REC domain (**Figure 2B**, inset). This interaction can also be observed in the recently reported structure of ARR1 alone (Zhou et al., 2024) (PDB ID: 8XAS), highlighting the relevance of the ARR1-REC and GARP interface.

Sakai et al. showed that ARR1 and ARR2 without the REC domain (termed ARR1ΔDDK and ARR2ΔDDK, respectively) exhibited much higher transactivation activities than ARR1 and ARR2, respectively (Sakai et al., 2000). Similar observation in ARR11 was also reported (Imamura et al., 2001). These results indicate that the REC domain suppresses the activity of the GARP DNA-binding domain, and that phosphorylation following cytokinin treatment alleviates this suppression. The structural data are consistent with this observation: (i) there is a tight interface between the REC and GARP domains, and (ii) the GARP α3* helix adopts a different conformation when bound to DNA (Zhou et al., 2024). In other words, because part of the GARP interface that binds DNA is also involved in the GARP-REC interdomain interaction, GARP must be set free for it to bind DNA. However, the molecular determinants of that event remain unresolved.

### The AHP1/ARR1-RG interface

**Figure 3:**
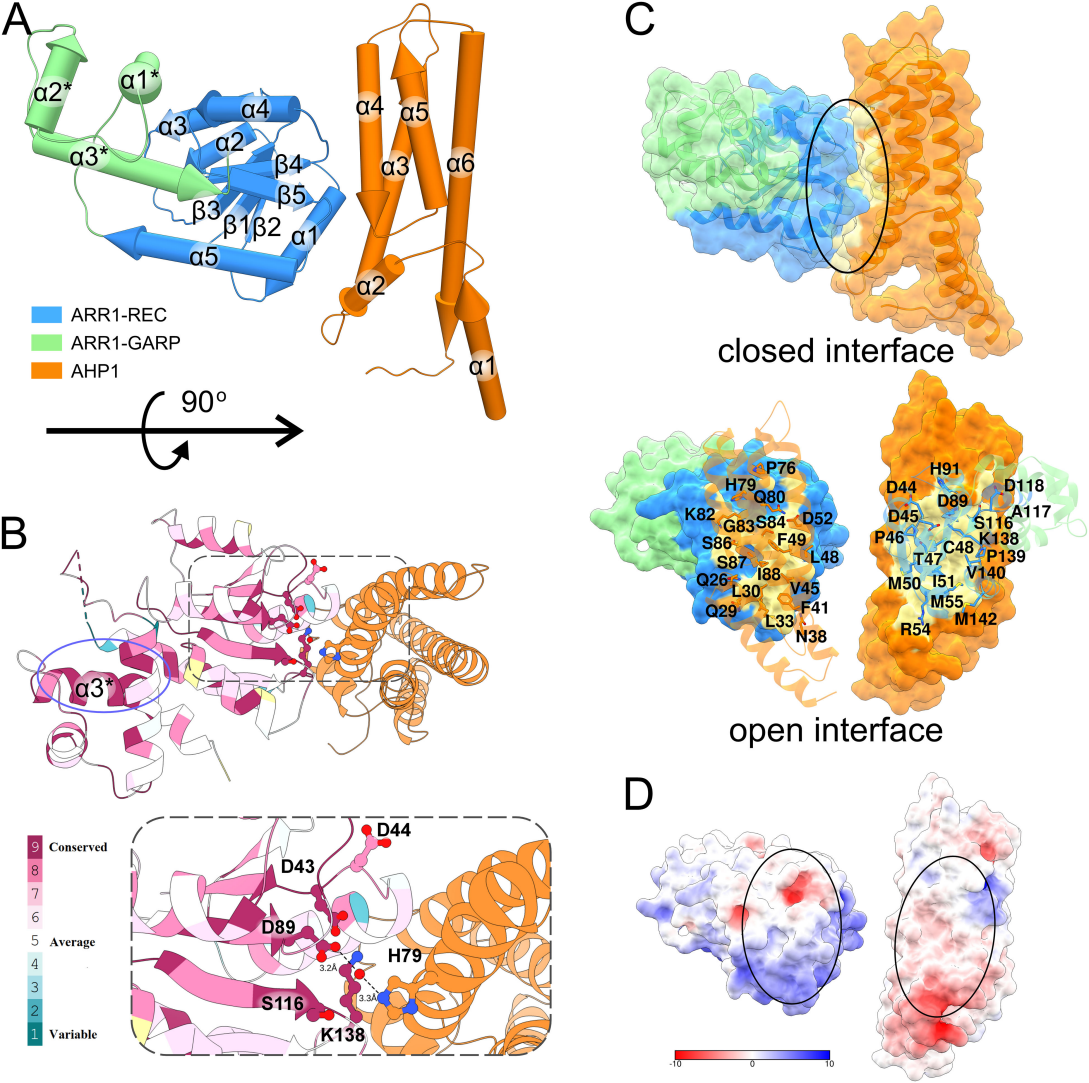
Structure of the AHP1/ARR1-RG complex. In **(A)**, the structure of the complex is shown in pipe-and-plank representation. The ARR1-REC-GARP structure is blue (REC domain) and light green (GARP domain) and the AHP1 structure is orange. In **(B)**, the conservation of type-B ARR is mapped onto ARR1-RG structure using Consurf web server with the alignment from Clustal Omega. The active site is highly conserved as depicted in the inset. AHP1 and ARR1 bind in such a manner that the two key residues, D89-ARR1 and H79-AHP1, are connected via a water molecule (red ball). The highly conserved GARP α3 helix is circled in violet. **(C)** shows the binding interface of ARR1-RG and AHP1 (upper image, black ellipse), and the residues involved in the interface in the artificially open interface (bottom image). **(D)** shows the interface colored by electrostatic potential (calculated by APBS webserver at pH 7.5).

AHP1 is composed of six helices with various lengths (from 3 to 10 turns), in which helices α3-α6 form a four-helix bundle (**Figure 3A**). The phosphorylatable His79 (Ruszkowski et al., 2013) in helix α4 is exposed towards the ARR1 recognition site, which is highly conserved among type-B ARRs (**Figure 3B**, **Supplementary Figure S1**). In the case of ARR1, it is composed of the catalytic Asp89 at the C-terminus of the β3 strand, the double Asp motif (43-44) in the loop between β1 and α1, Ser116 at the C-terminus of β4, and Lys138 in the loop between β5 and α5. The two Asp residues are involved in coordinating Mg^2+^ ion, essential for the phosphorylation reaction, as similarly observed in the REC domain of CRE1 (Tran et al., 2021). In bacterial RRs, the position corresponding to Ser116 is occupied by a highly conserved Thr residue, which is proposed to contribute to the Y-T conformational switch upon RR phosphorylation (Zhu et al., 1997; Birck et al., 1999; Hastings et al., 2003). The position of Lys138 suggests its role in coordinating the phosphate moiety once the RR is phosphorylated. Interestingly, the equivalent residue has been reported to participate in the domain swapping dimerization of Spo0A upon phosphorylation (Lewis et al., 2000) via Lys-Pro *cis-trans* isomerization.

ARR1-RG binds AHP1 with a 1:1 stoichiometry (**Figure 3C**) through a relatively small interface of 875 Å^2^. The ratio of buried surface area over total surface area (BSA/TSA) for both ARR1-RG and AHP1 are less than 0.1. The interface involves up to fourteen hydrogen bonds (depending on the complex in the ASU) and three salt bridges. These interactions are brought about by up to nine residues from ARR1-RG (Asp44, Asp45, Thr47, Cys48, Arg54, Asp118, Lys138, Arg141, and Met142), of which Asp44 and Lys138 belong to the conserved active site. From the AHP1 side, there are up to 11 residues (Ser25, Gln26, Gln29, Leu33, Asp35, Gln80, Lys82, Gly83, Ser84, Ser87, and Arg101). Eight of these residues are identical among AHP1-5 (**Supplementary Figure S2**).

### ARR1-RG is able to bind AHP1-5 with a twofold preference towards AHP1

We investigated the ARR1 capability to interact with AHPs because such data are not available in the literature. To this end, we produced AHP1-5 as well as a biotinylated variant of ARR1-RG and employed biolayer interferometry (BLI). During the experiments, we observed that the ARR1-AHP interactions exhibited a rapid association, quickly reaching a plateau (**Supplementary Figure S3**). Thus, binding affinities were calculated using two methods: fitting the binding kinetics and fitting the steady-state data. While steady-state fitting only provides the binding affinity (K_D_), it lacks information about the association rate (*k*_a_) and dissociation rate (*k*_dis_). Notwithstanding, both fitting methods yielded consistent binding affinities for each interaction (**Table 2**).

**Table 2:** Binding affinities of ARR1-RG and AHP1 to AHP5.

ARR1-RG with	K_D_ (nM)	K_D_ error (nM)	Steady state K_D_ (nM)	K_a_ x10^5^ (M^-1^s^-1^)	K_a_ error x10^3^ (M^-1^s^-1^)	K_dis_ x10^-2^ (s^-1^)	K_dis_ error x10^-4^ (s^-1^)
AHP1	94	0.335	94	4.44	1.28	4.23	0.86
AHP2	160	0.612	160	5.73	1.72	9.13	2.13
AHP3	174	0.579	175	5.22	1.37	9.17	1.82
AHP4	166	0.704	170	3.60	1.19	6.00	1.35
AHP5	152	0.639	150	5.88	2.20	8.90	2.64

The binding affinity was calculated from kinetic data and steady state data. Each measurement was repeated at least twice.

The efficient signaling balances the transduction rate with specificity of protein-protein interactions, which themselves can be classified as permanent or transient. Permanent interactions are characterized by strong binding affinity (below low-nM K_D_), while transient interactions produce bound-unbound equilibrium, with K_D_ values of hundreds nM but not µM (Perkins et al., 2010). AHP1-5 interact with ARR1-RG with moderate binding affinities in the range of 94-174 nM. Among these, the strongest binding affinity (94 nM) was demonstrated by AHP1. AHP2-5 show more or less similar binding affinities, characterized by K_D_ of 160, 174, 166 and 152 nM, respectively, and also fall in the regime of strong transient interactions. Additionally, we deduced the association and dissociation rates (*k*_a_ and *k*_dis_, respectively, **Table 2**). The data revealed that the association rates are similar for AHP1-5, ranging from 3.60 x 10^5^ to 5.88 x 10^5^ M^-1^ s^-1^. Generally, protein-protein association rates range from 10^3^ to 10^9^ M^-1^ s^-1^ (Alsallaq and Zhou, 2008), and are mostly limited by diffusion and the proper orientation of the two proteins with respect to one another. Therefore, without other enhancing factors, such as electrostatics, this association rate is limited to 10^5^ - 10^6^ M^-1^ s^-1^ (Alsallaq and Zhou, 2008). The measured *k*_a_ values for the AHP/ARR1 interactions can be classified as marginally fast, which is clearly illustrated by the steep binding curve (**Supplementary Figure S3**). Moreover, analysis of the surface electrostatic potential distribution at the AHP1/ARR1 interface indicated only minor patches of positive-negative matching pairs (**Figure 3D**). This suggests that the hydrophobic effect, short-range nonbonding interactions, and shape matching are the major factors responsible for AHP/ARR1 recognition.

Most importantly, the main difference in binding affinities results from the dissociation rates, which span a range of 4.23 - 9.17 x 10^-2^ s^-1^, with AHP1 dissociating the slowest. Interestingly, there are 15 identical residues out of 24 that contribute to the interfaces in AHP1-5 (**Supplementary Figure S2**). From the alignment, one can also see that the interfaces involved are made of several linear motifs of 3-8 residues. Although each linear motif likely provides a weak affinity, combining several of them ensures sufficient binding affinity as well as improves the specificity of the protein-protein interactions. In addition, none of the substitutions in other AHPs (**Supplementary Figure S2**), which per analogy should be involved in the recognition, appear to drive the repulsion, which agrees with our BLI experiments.

Unfortunately, in the current setup, we were not able to test the behavior of phosphorylated AHPs, because we were not able to produce *At*CRE1/AHK4, AHK2 or AHK3. On the other hand, while it is possible to produce a catalytically active intracellular part of CRE1 from *Medicago truncatula* and use it to phosphorylate *Mt*HPt1 (Ruszkowski et al., 2013), *Mt*CRE1 did not phosphorylate AHPs to a measurable extent (not shown). An explanation could be provided by the fact that Asp-His phosphorylation is labile, which makes the measurements complicated. Obviously, the binding of ARR1-RG to unphosphorylated AHP1-5 does not contradict the direction of the signaling cascade. However, it opens a potential possibility that in the absence of phosphorylation, AHPs may act as molecular sinks, temporarily sequestering ARR1.

### AHP1/ARR1-RG *vs* AHK5-REC/AHP1

**Figure 4:**
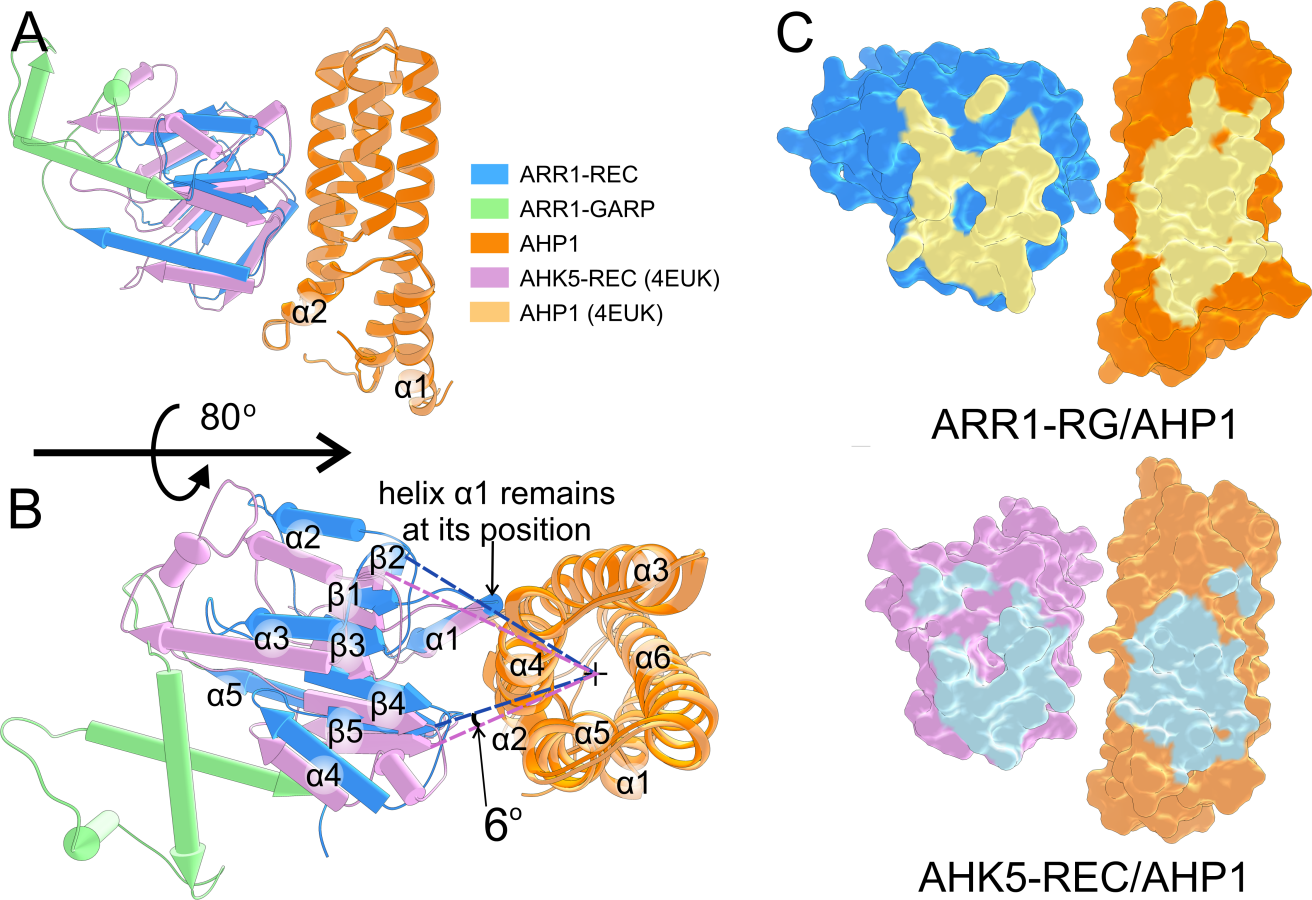
Comparison of the AHP1/ARR1-RG and AHK5-REC/AHP1 complexes. **(A)** shows the overview of the superposition between the ARR1-RG/AHP1 complex and the AHK5-REC/AHP1 complex (PDB: 4EUK); AHP1 subunits were superposed. The two AHP1 molecules are highly similar (RMSD of 0.69 Å). The REC domains bind AHP1 at a slightly different angle (about 6°) as shown in **(B)**. The binding interface of the two complexes is shown in **(C)**.

Our AHP1/ARR1-RG structure is the first one that illustrates this part of cytokinin signal transduction pathway, i.e., the complex of an AHP (or HPt) protein with a plant RR. The other structure in the PDB involves the upstream elements, showing the AHK5-REC/AHP1 complex (PDB: 4EUK); we use the notation to represent the order in the signaling cascade. The structural superposition of the two structures using MatchMaker within UCSF Chimera (Meng et al., 2006) (by pairing two AHP1 subunits) results in RMSD of 0.69 Å (across 145 pruned atom pairs). The major discrepancy is in the slightly different positions of α1 and α2, while the structure of the four-helix bundle is similar (**Figure 4A**). However, the REC domains of ARR1 and AHK5 are bound to AHP1 at slightly different positions. Considering the four-helix bundle AHP1 as a cylinder, the β-sheet of AHK5-REC (perpendicular to the cylinder) is rotated about 6° toward the α5 helix (**Figure 4B**). The ARR1 REC α1 helix is positioned similar to the equivalent helix in AHK5-REC, but the rest is shifted by ~2 Å further from the α4-α3 helices (**Figure 4B**). Thus, the two binding interfaces are slightly different (**Figure 4C**). Nonetheless, it is remarkable that despite the REC domains of ARR1 and AHK5 share low sequence identity (22%), their fold is highly similar, with the RMSD of 1.06 Å across 94 pruned atom pairs.

In AHK5-REC/AHP1, ten hydrogen bonds contribute to the binding, but there are no salt bridges at the interface. The H-bonds involve ten residues of AHP1 (Lys82, Gln26, Gln80, Ser84, Ser83, Ser87, Asn38, Asp35, Gln29, and Gln32) and seven residues of AHK5-REC (Ala885, Val908, Asn786, Asn789, Ser795, Gln799, and Leu910). A general note for both complexes is that the number of residues involved in the interface is larger for AHP1 than for the REC domain. REC, which contributes fewer interface residues by sheer number, contains nearly all the residues that are essential for the phosphotransfer reaction. Thus, it seems likely that variations in the AHPs sequence at the interface have a greater effect on the binding affinity and selectivity toward ARRs, which themselves control the kinetics.

The interface scores calculated by PDBePISA (Krissinel, 2010) for AHP1/ARR1-RG and AHK5-REC/AHP1 are 1.00 and 0.66, respectively. These results are consistent with the longer distance between AHK5-REC and AHP1. The small difference in binding may also be attributed to the presence of BeF_3_^-^, which mimics the phosphorylation state of REC in the AHK5-REC/AHP1 complex structure (4EUK), potentially leading to different binding. However, it is worth noting that the distance between the phosphorylatable His and Asp (Nε-Oδ atoms) remains similar, being equal to 6.45 Å and 6.33 Å in AHP1/ARR1-RG and AHK5-REC/AHP1, respectively.

### AHP and HPt oligomerization state

**Figure 5:**
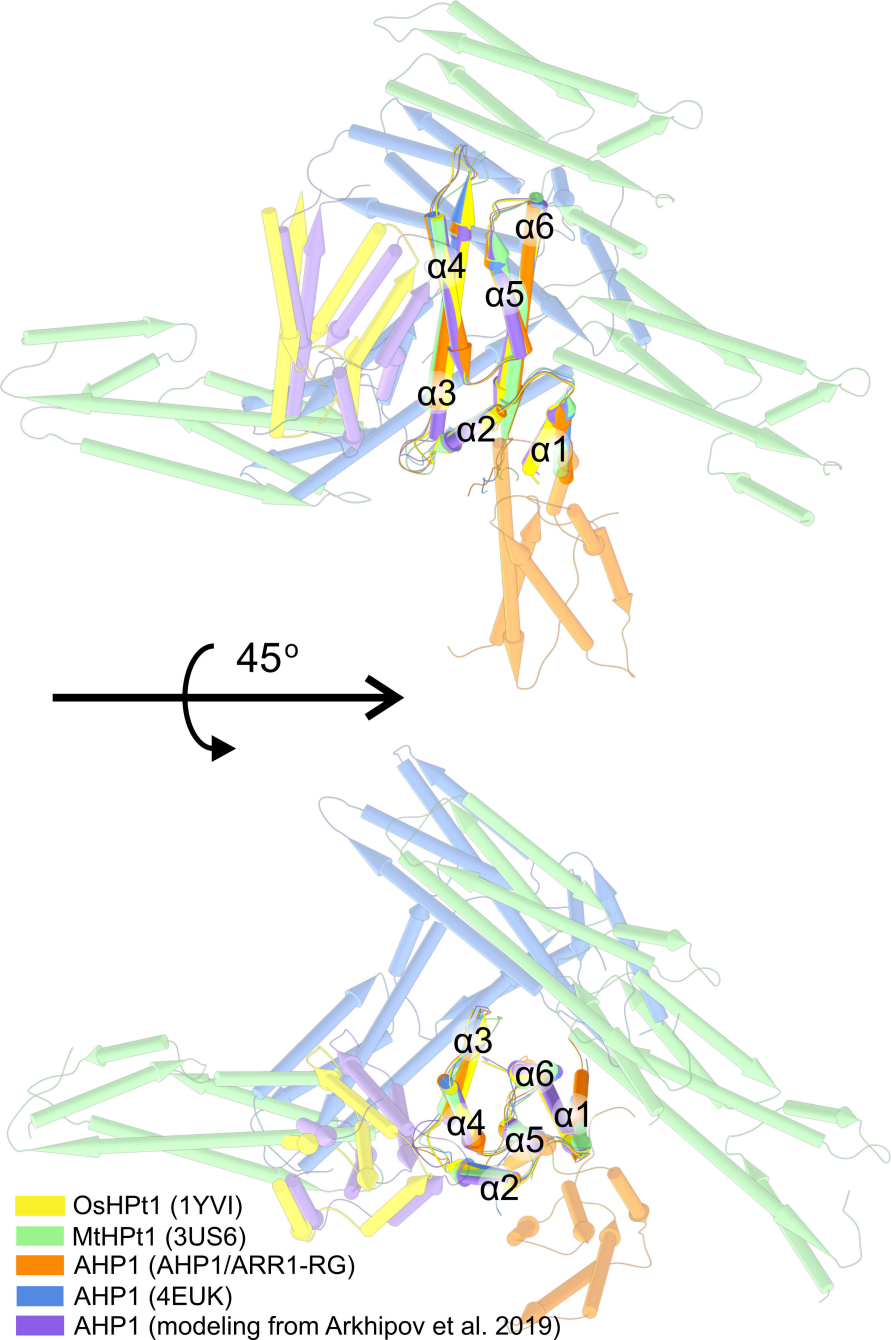
The superposed structures of AHP1, MtHPt1, and OsHPt1. In silico model of dimeric AHP1 from Arkhipov et al. (2019) is in purple, the model was based on OsHPt1 structure (PDB ID: 1YVI, yellow), explaining the resemblance. AHP1 and HPt1 structures were aligned and symmetry-mate molecules were shown to compare all possible contact interfaces of AHP1 and HPt1 proteins.

Since AHPs (and HPts) heterooligomerize with the REC domains of ARRs and histidine kinases, we were intrigued whether they could also homooligomerize. The RR phosphotransferases in prokaryotes were shown to form dimers (Varughese et al., 1998), but it is difficult to trace back the evolution of AHPs. It has been suggested that AHPs evolved from proteins other than the homodimeric DHp domain of histidine kinase (Capra and Laub, 2012). Consistently, there has been a debate regarding plant AHP (or HPt) oligomerization, and attempts have been made to model the structure of the homodimer and heterodimer of AHP1, AHP2, and AHP3 *in silico* (Arkhipov et al., 2019). In the absence of other and more convincing data, we analyzed the possibility of AHP dimerization by comparing crystal contacts in four experimental structures, including *Mt*HPt1 (PDB ID: 3US6), *Os*HPt1 (1YVI, 2Q4F), and two structures containing AHP1 (our structure and PDB ID: 4EUK) with the model presented by Arkhipov et al. The rationale behind our approach was that if a physiologically relevant AHP/AHP (or HPt/HPt) interface existed, it would definitely make up one of the protein-protein contacts in the crystal lattice. On the contrary, our analysis revealed that the AHP-AHP and HPt-HPt contacts in the crystal lattices were created mostly by weak interactions, and (most importantly) were different in every case (**Figure 5**). For instance, in our structure, the two AHP1 molecules (within the ASU) interact via the surface of α2-α5 towards α1-2-5-6 of the second molecule. Meanwhile, in 4EUK, the second AHP1 molecule is perpendicular to the first. In *Mt*HPt1, these interactions occur via loops. Notably, there are two subunits in the *Os*HPt1 ASU. However, the PBDePISA (Krissinel, 2010) analysis suggested that the contact is unlikely to be biologically relevant, attributing it purely to crystal packing. The model of dimeric AHP1 in the work of Arkhipov et al., 2019 was based on this *Os*HPt1 structure, thus, shares a very similar interface. However, in addition to the crystal contacts being different in every case, the protein-protein contacts are never related by a (non-screw) two-fold axis (either crystallographic or non-crystallographic), which is a requirement for a biologically relevant dimerization. Altogether, AHP/AHP (and HPt/HPt) interactions are most likely the effect of crystal packing and are biologically irrelevant, whereas existing experimental data only support the monomeric states of free AHP1 and other plant HPts.

## Conclusion and perspective

In this work, we presented the first structure of the ARR1-RG and AHP1 complex. The structure revealed a binding interface that includes several residues that are conserved among AHPs. The binding interface has an area of less than 1000 Å^2^, which is consistent with the transient nature of the AHP1/ARR1 interaction. We also measured the interactions between ARR1-RG and AHP1, AHP2, AHP3, AHP4, and AHP5, which are all five active AHPs in *A. thaliana*. The obtained K_D_ was 94 nM for AHP1 and 150-175 nM for AHP2-5. The binding measurements corroborated the dynamic character of AHP/ARR1 recognition in the signaling cascade, which includes (i) fast association increasing the binding affinity, (ii) moderate binding affinity, and (iii) rapid dissociation, providing the necessary rate for signal transduction. Multiple studies demonstrated that an individual histidine kinase could employ specific sets of AHPs and ARRs in response with certain stimuli (Urao et al., 2000; Hwang and Sheen, 2001; Mira-Rodado et al., 2012; Mira-Rodado, 2019). Our results suggest that ARR1 is a universal RR in MSP systems, as it is able to interact with all AHPs with only a two-fold preference for AHP1. This result is interesting in the context of the transient nature of the signaling cascade, indicating that non-phosphorylated AHPs could also bind (and sequester) ARR1. Finally, we postulate that AHP and HPt proteins do not form homodimers, as previously suggested, because the AHP1-AHP1 interfaces in the available crystal structures are all different and not related by a two-fold symmetry.

The intriguing question now is how phosphorylation of ARR1 releases the GARP DNA-binding domain. In prokaryotic TCSs, phosphorylation triggers a large conformational change, including a change in the oligomerization state, leading to activation of the DNA-binding domain (Lewis et al., 2000; Leonard et al., 2013; Boyaci et al., 2016). Theoretically, ARR1 could form swapped dimers, similar to what we reported for the REC domain of CRE1 (Tran et al., 2021); however, this speculation is not supported by any experimental data. In another scenario, the phosphorylation could induce a conformational change in the flexible, unstructured linker between REC and GARP, thus dragging out the GARP domain. Such a conformational change has been observed in *S. aureus* VraR RR, which also dimerizes upon phosphorylation (Leonard et al., 2013). In this case, the helix connecting the DNA-binding domain with REC was untwisted (within 10 residues), pulling out the DNA-binding domain. However, the corresponding linker in ARR1 is significantly longer (77 residues) and appears highly flexible, making it unlikely to exert sufficient force to displace the GARP domain in a similar manner.

Another possibility relies on allosteric regulation, for instance involving Y-T coupling. Both residues are quite conserved (the corresponding positions in ARR1 are Tyr135 and Ser116); Thr/Ser occupies the β4 C-terminus, and Tyr (in some cases Phe) lies in the center of the β5 strand. According to Y-T coupling, phosphorylation tunes the orientation of Thr/Ser, which in turn reorients Tyr/Phe, causing further rearrangements in the 3D structure. Examples include unswapping of non-phosphorylated Spo0A dimer to a phosphorylated monomer (Lewis et al., 2000), and dimerization upon phosphorylation in the FixJ receiver domain (Birck et al., 1999). There are several other examples of allosteric control such as NtrC, PhoB (Fiedler and Weiss, 1995), and CheY (Zhu et al., 1997). Another allosteric mechanism in RRs, β5-T coupling, is related to RscB (Casino et al., 2018). It is noteworthy that in RscB, the REC and DNA-binding domains are connected via a 24-residue linker.

We cannot exclude the scenario in which GARP dissociation involves the binding of supporting protein(s). As summarized in a review describing transcription factor complex formation (Leuendorf and Schmulling, 2021), ARR1 can directly interact with several other proteins, including ARR12 and EIN3. The formation of the complex can regulate the transcriptional activity of either transcription factor, possibly contributing to the cross-talk between signaling cascades. In a recent study, ARR1 was shown to be SUMOylated at Lys236 (Kang et al., 2024). This SUMOylation affects ARR1 activity as a transcriptional regulator, which is consistent with the fact that Lys236 marks the N-terminus of the GARP domain in our structure.

The aforementioned scenarios do not necessarily contradict each other but due to the lability of phosphorylated histidine and aspartate, capturing the structures at different signaling steps is challenging. Nonetheless, this study brings us a step closer to fully dissecting plant MSP. To date, the structures of several elements of this pathway have been revealed. Regarding the first element, sensory HK, the structures of the cytokinin-binding domain (Hothorn et al., 2011), and the receiver domain (Tran et al., 2021) have been reported for CRE1. The complex of REC domain in the CRE1 homolog (AHK5) and AHP1 (Bauer et al., 2013) presented the snapshot of the first phosphotransfer. Structures of the second element (AHP or HPt), are available for AHP1 (Bauer et al., 2013) and AHP2 (PDB code: 4PAC), among several examples for other plant species. As for the third element (RR), the DNA binding domains of ARR10 (Hosoda et al., 2002) and ARR1-RG (Zhou et al., 2024) have been published. Finally, the complex GARP/DNA has also been revealed (Zhou et al., 2024). Therefore, the current study introduces an additional bridge between AHP1 and ARR1, showing the architecture of the complex. In addition to answering the aforementioned question about the GARP release mechanism, the biggest missing piece of the puzzle is the structure of the entire CRE1. But that is not all, since the MSP can also involve other biological partners that introduce extra layers of complexity into signaling. SUMOylation of ARR1 is one example (Kang et al., 2024), and the other is S-nitrosylation of AHP1, which decreases the phosphotransfer rate (Feng et al., 2013). This added complexity may play a role in facilitating hormone cross-talk in plants. The inclusion of more elements in the signaling cascade enables more precise regulation of the signaling and more effective management of the signaling direction. All this makes the structural characterization of cytokinin MSP incredibly interesting, challenging, and far from complete.

## Materials and methods

### Cloning, overexpression, and purification

The coding sequences for AHP1 and ARR1 were retrieved from UniProt (IDs: Q9ZNV9 and Q940D0 respectively). The DNA fragments were amplified using Platinum SuperFi II Master Mix and *A. thaliana* cDNA as the template. The cDNA was prepared from the plant rosette using the GeneMATRIX Universal RNA purification kit (EURx) and later converted to cDNA using SuperScript TM III Reverse Transcriptase (Invitrogen). The primers list for all DNA manipulations is provided in the **Supplementary Table S1**. The full-length *AHP1* was cloned into vector pMCSG53 (Midwest Center for Structural Genomics) according to the ligase-independent cloning protocol (Kim et al., 2011), which was then used to transform *E. coli* BL21 Gold (Agilent) competent cells. For ARR1, the domain boundaries were estimated using the AlphaFold model (Mirdita et al., 2022), and the sequence including the receiver domain and the GARP motif was used. The ARR1 constructs for structural studies and BLI measurements spanned residues 38-296 and 1-296, respectively. The cloning steps were the same as for AHP1.

The *E. coli* culture was cultivated in LB medium supplemented with 150 µg/mL ampicillin at 37°C until OD reached 0.8. The culture was cooled down to 18°C and the protein expression was induced using 0.5 mM of isopropyl β-D-1-thiogalactopyranoside (IPTG). After 18 h, the cells were collected by centrifugation at 5000 × *g* for 20 mins and suspended in 35 mL of cold Binding buffer (50 mM HEPES-NaOH pH 7.5, 500 mM NaCl, 20 mM imidazole, 1 mM TCEP) and stored at -80°C.

After thawing, the cells were sonicated in an ice bath with the pulse mode 4 s ON: 26 s OFF for 5 mins of probe working time. The sample was then centrifuged at 27000 × *g* for 30 mins at 4°C and the supernatant was collected. It was loaded into pre-equilibrated His trap HP Ni-NTA resin (GE Healthcare) and washed 5 times using the Binding buffer. The proteins were eluted using Elution buffer (50 mM HEPES-NaOH pH 7.5, 500 mM NaCl, 400 mM imidazole, 1 mM TCEP). For AHP1, the eluted protein was dialyzed overnight at 4°C against Dialysis buffer (50 mM HEPES-NaOH pH 7.5, 500 mM NaCl, 1 mM TCEP) using 3 kDa cutoff Snakeskin dialysis tubing (Thermo Fisher). Simultaneously, 500-µL aliquot of TEV (2mg/mL) was added to the dialyzing tube to remove His-tag. The next day, the protein solution was applied to the Ni-NTA column to capture free His-tag. For ARR1-RG, in the first purification attempt, we observed that TEV protease was not able to cleave the His-tag, implying that probably the TEV cleavage site was not accessible. Thus, later on, ARR1-RG was purified in one step using His trap HP Ni-NTA resin. Then, the two proteins were mixed in the molar ratio of 1 ARR1: 1.5 AHP1. The mixture was concentrated to 2 mL volume and applied onto the Superdex 200-16-60 column in SEC buffer (25 mM HEPES pH 7.5, 50 mM NaCl, 100 mM KCl, 1 mM TCEP) in the AKTA FPLC system. Fractions corresponding to the complex were collected and concentrated to above 30 mg/mL for crystallization.

For BLI experiment, AHP2 (Uniprot ID: Q9ZNV8), AHP3 (Q9SAZ5), AHP4 (F4J1I8) and AHP5 (Q8L9T7) were prepared similarly to AHP1, except for AHP4, for which only cDNA from mature leaf gave the product. AHP3, AHP4 and AHP5 were cloned into the pMCSG53 vector while AHP2 was cloned into pMCSG48 containing NusA fusion at N-terminus; the final protein products are preceded by SNA linker in each case. The same purification protocol was used, starting with the 1^st^ His trap, followed by the overnight TEV digestion to remove the His-tag or His-tag-NusA fusion. Then, the 2^nd^ His trap was done to capture the cleaved tag/fusion, and the proteins were purified with SEC. All proteins were purified using the same buffers, except for AHP4, for which the pH was adjusted to 7.0 to stay away from its pI (7.7). For ARR1-RG, the coding sequence was also cloned into the pMCSG62 vector, which provides the Avi-tag that could be biotinylated by BirA protein. BirA coding sequence was cloned separately to pRSF vector, which introduces kanamycin resistance. Finally, ARR1-REC-GARP and BirA were co-expressed in the *E. coli* BL21 Gold strain. The culture and purification conditions were similar as for ARR1-RG in vector pMCSG53.

### Crystallization

The crystallization screening was set up using the vapor diffusion method in 96-well plates. Several screens were tested, including Index (Hampton Research), BCS (Chaikuad et al., 2015) (Molecular Dimensions), and Morpheus (Gorrec, 2009) (Molecular Dimensions). Various protein concentrations were tested together with varying volume ratios of protein: reservoir were used (1:1, 2:1, 1:2). Several conditions yielded star-shape or even sea-urchin-like crystals. Only in the Morpheus screen, there were some more promising crystal morphologies. Several attempts to optimize the crystals out of these conditions failed as crystallizations were not repeatable. Finally, several copies of the Morpheus screen with the same protein concentration and same ratio were set up. Interestingly, diffraction-quality crystals appeared in different conditions in different plates despite the same set-up. The best diffracting crystal was grown in the condition G11 (Gorrec, 2009), which includes 20 mM Sodium formate, 20 mM ammonium acetate, 20 mM sodium citrate tribasic, 20 mM potassium sodium tartrate, 20 mM sodium oxamate; 100 mM Tris (base) BICINE pH 8.5; 20% glycerol, 10% PEG 4000. The protein concentration was 32.5 mg/mL and the ratio of protein: reservoir was 1:1. No additional cryoprotectant was used while harvesting the crystal and flash vitrifying in liquid nitrogen.

### Data collection and processing, structure solution, and refinement

The X-ray diffraction data was collected at the EMBL P13 beamline of the PETRA III storage ring at DESY Hamburg (Cianci et al., 2017). The diffraction images were processed using XDS (Kabsch, 2010). The resolution was truncated anisotropically with the Staraniso (Tickle et al., 2018) server and reached 2.9 Å in the best direction. The data statistics are presented in **Table 1**.

The crystal structure was solved by molecular replacement using PHASER (Mccoy et al., 2007). The model of AHP1 was taken from the PDB (ID: 4EUK). The model for ARR1 was generated by AlphaFold (Jumper et al., 2021). Only ARR1-REC (38-158) was taken as the model for molecular replacement. PHASER was run with the search for 2 molecules of AHP1 and 2 molecules of ARR1-REC based on the calculation of Matthews coefficient. However, the placement of the 2^nd^ ARR1-REC molecule was not correct. Thus, an initial model of the complex including one AHP1 and one ARR1-REC was created. Molecular replacement was repeated in the search for two complexes. Afterward, the three helices of GARP were placed manually into the electron density map. Notably, the entire GARP domain was predicted by Alphafold to occupy a completely different place.

The structure was refined through several rounds of manual model corrections in Coot (Emsley et al., 2010), intertwined with automatic refinement in Phenix.Refine (Afonine et al., 2012). The final model reached R-work and R-free of 19.13% and 22.86%, respectively; refinement details are listed in **Table 1**.

### Bio-layer interferometry

The interaction measurements were set up as follows. The biotinylated ARR1-REC-GARP was the ligand immobilized onto the Octet SA Biosensors while AHPs were analytes. The kinetic buffer was 20 mM HEPES/NaOH pH 7.0, 50 mM NaCl, 50 mM KCl, 0.5 mM TCEP, 0.5% BSA, and 0.05% Tween 20. First, the optimal loading conditions were tested, in which the concentration of ARR1-RG was varied from 5 µg/mL to 25 µg/mL and the loading time tested was 300 s and 600 s. The analyte concentration was 500 nM with the association and dissociation times being 120 s and 180 s, respectively.

For the kinetic study, ARR1-RG was loaded for 300 s using a 10 µg/mL solution. The association and dissociation times were both 180 s. The concentration of AHPs was varied from 12.5 µM to 300 µM in two-fold dilution series, organized from the lowest to the highest concentration. The initial tests showed that AHPs associate very fast and could fully dissociate from ARR1-RG. The association time was also prolonged to 180 s to enable reliable inferring of K_D_ from the steady-state analysis. A long dissociation was used instead of regenerating the sensor owing to the nearly irreversible binding of ARR1 to the sensor. The instrument temperature was set to 25°C for all measurements. Regarding the quality of the fitting, all fits gave R^2^ values of 0.99 and the X^2^ ranged from 0.18 to 1.51 (not shown).

### Other software

All proteins alignments were perform using Clustal Omega (Madeira et al., 2024) and the conservation of the sequence is mapped into protein structure using Consurf (Ashkenazy et al., 2016). All the proteins visualization was done in Chimera (Pettersen et al., 2004) and ChimeraX (Meng et al., 2023). The electrostatic potential surface was calculated using APBS server (Jurrus et al., 2018).

## Data Availability

The final model of the AHP1/ARR1-RG complex was deposited in the PDB (http://www.wwpdb.org/) together with the structure factors, PDB ID: 9H6E. Raw X-ray diffraction data were deposited in the Macromolecular Xtallography Raw Data Repository (MX-RDR): complex ARR1-AHP1, https://doi.org/10.60884/I1X67S.
