# Supplementary material for: ARR1 and AHP interactions in the multi-step phosphorelay system
Source: Front Plant Sci. 2025 Feb 27;16:1537021. doi: 10.3389/fpls.2025.1537021 (PMC11903765; doi:10.3389/fpls.2025.1537021)
Supplement: Supplementary file 1 [file DataSheet1.docx]

**Supplementary data**

Structural Basis of AHP1 and ARR1 Interaction

Linh H. Tran ^1^, Milosz Ruszkowski ^1^

^1^ Institute of Bioorganic Chemistry, Polish Academy of Sciences, Poznan, Poland

*Correspondence:

Milosz Ruszkowski

Institute of Bioorganic Chemistry,

Polish Academy of Sciences,

Noskowskiego 12/14

61-704 Poznan, Poland

E-mail: [mruszkowski@ibch.poznan.pl](mailto:mruszkowski@ibch.poznan.pl)

**List of supplementary tables and figures**

Supplementary Figure S1: Sequence alignment of type-B ARRs.

Supplementary Figure S2: Sequence alignment of AHPs.

Supplementary Figure S3: Bio-layer interferometry (BLI) kinetic binding curves of ARR1-RG and AHPs.

Supplementary Table S1: Sequences of primers used for cloning.


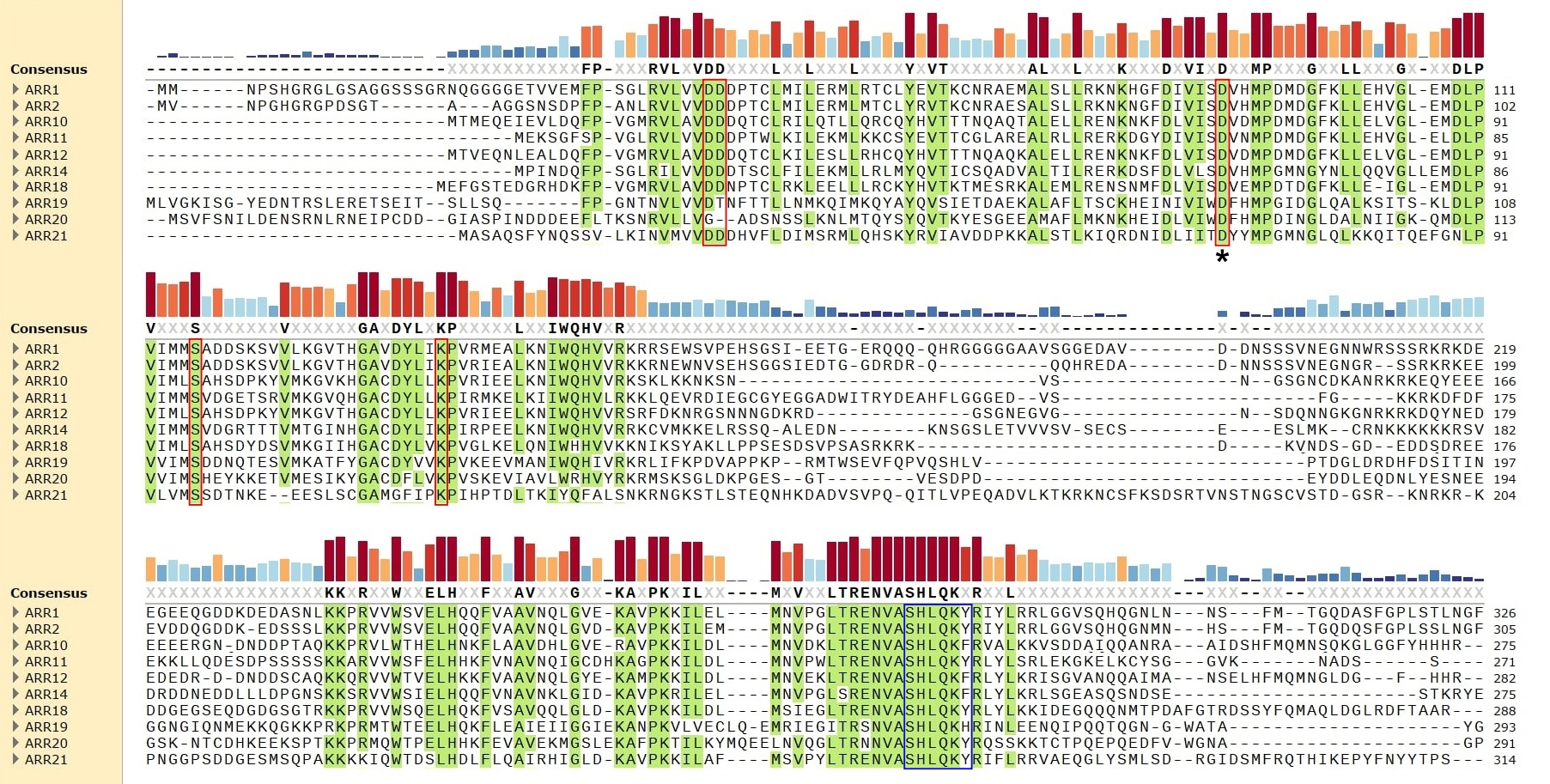


**Figure S1**: Sequence alignment of all type-B ARRs proteins with the residues having consensus ≥ 70% marked in green. The full-length sequences were aligned but only the REC domain and the GARP domain are shown here. The catalytic center residues are marked in red rectangular and the DNA bind sequence is marked in blue rectangular. The phosphorylated Asp (Asp89 in ARR1) is marked with an asterisk.


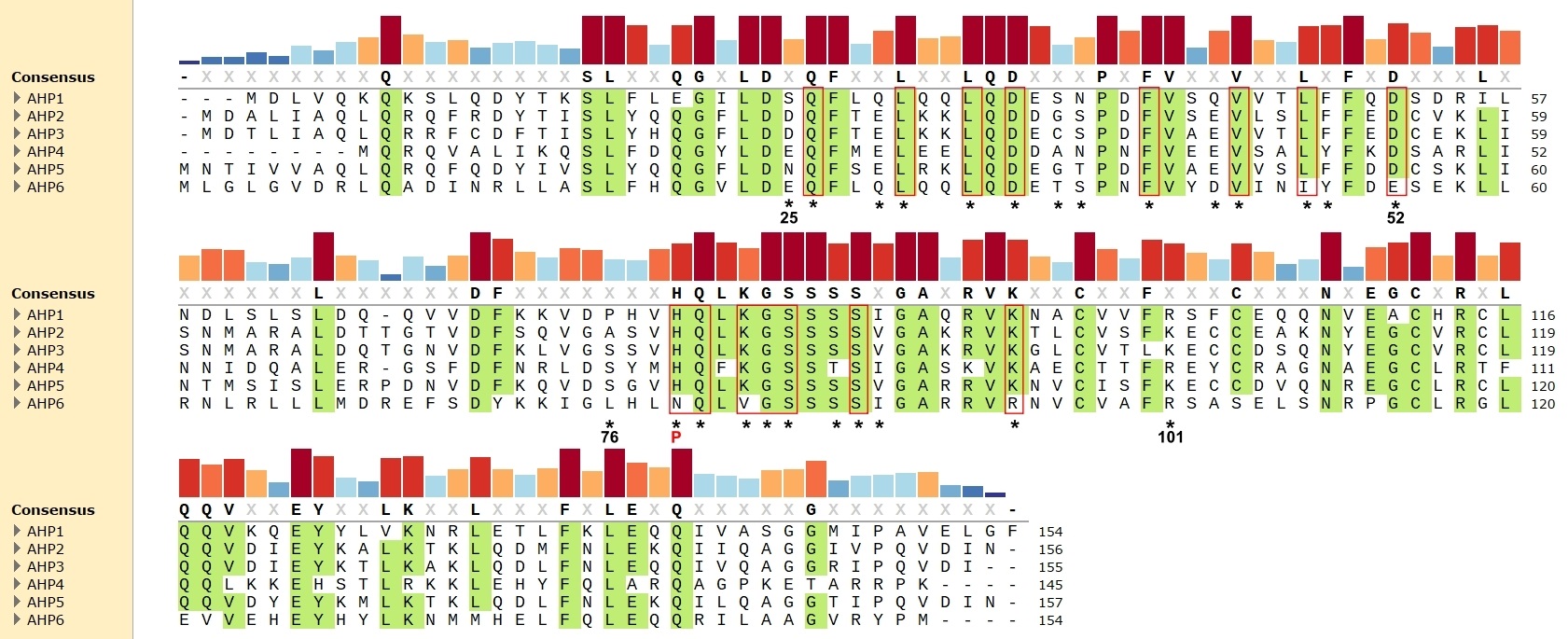


**Figure S2**: Sequence alignment of all AHP proteins with the residues having consensus ≥ 80% marked in green. Those residues involving in the interface between ARR1-RG and AHP1 are marked with asterisk and those identical residues among AHP1-5 are marked additionally in red square. Residue numbering corresponds to AHP1. The phosphorylated histidine (His79 in AHP1) is marked by capital red “P”. AHP6, which lacks the phosphorylable histidine and acts as the signaling inhibitor, has been included in the alignment for reference.


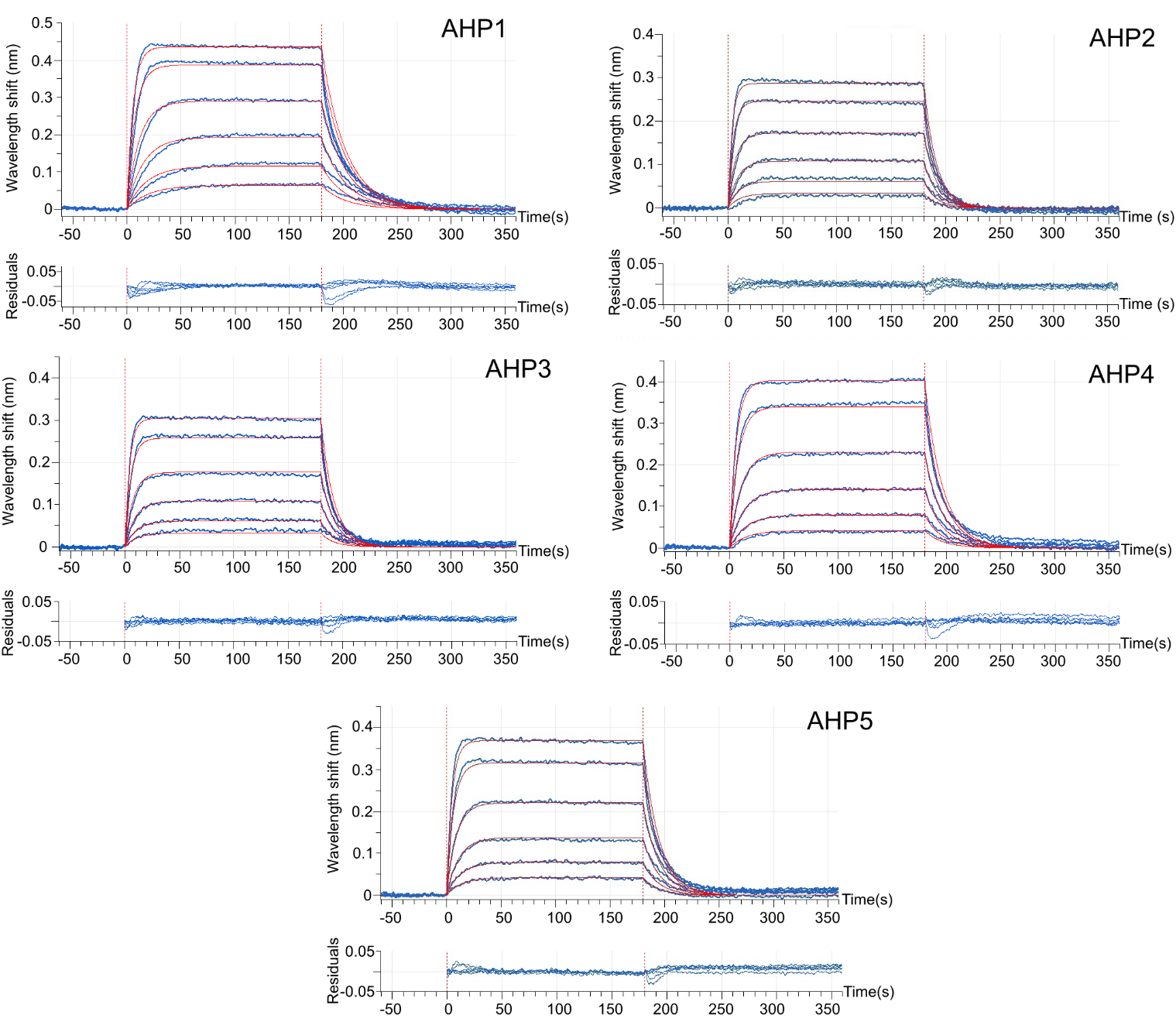


**Figure S3**: Bio-layer interferometry (BLI) curves (baseline, association, dissociation, separated by the red vertical lines). Biotinylated ARR1 was immobilized on the Octet SA Biosensors using 10 µg/mL solution for 300s. The concentration of AHPs varied from 300 µM to 12.5 µM (by two times dilution). An empty sensor (no ligand) was used in parallel to monitor and subtract the non-specific binding of analytes to the sensor. A zero concentration of analytes was also used to observe and subtract buffer interferences from the buffer.

**Table S1:** Primers for cloning

| **Primer name** | **5’ -> 3’ sequence** | **Note** |
| --- | --- | --- |
| AHP1-F | TACTTCCAATCCAATGCCATGGATTTGGTTCAGAAGCAGAAGAGTTT |  |
| AHP1-R | TTATCCACTTCCAATGTTAAAATCCGAGTTCGACGGCCGG |  |
| AHP2-F | TACTTCCAATCCAATGCCATGGACGCTCTCATTGCTCAGCTT |  |
| AHP2-R | TTATCCACTTCCAATGTTAGTTAATATCCACTTGAGGAACTATACCAC |  |
| AHP3-F | TACTTCCAATCCAATGCCATGGACACACTCATTGCTCAGTTACAGA |  |
| AHP3-R | TTATCCACTTCCAATGTTATATATCCACTTGAGGGATTCTACCACCA |  |
| AHP4-F | TACTTCCAATCCAATGCCATGCAGAGGCAAGTGGCACTCATCAA |  |
| AHP4-R | TTATCCACTTCCAATGTTACTTGGGCCTACGTGCTGTCTCC |  |
| AHP5-F | TACTTCCAATCCAATGCCATGAACACCATCGTCGTTGCTCAGTT |  |
| AHP5-R | TTATCCACTTCCAATGTTAATTTATATCCACTTGAGGAATTGTACCTCCA |  |
| ARR1-1-F | TACTTCCAATCCAATGCCATGATGAATCCGAGTCACGGAAGAG |  |
| ARR1-38-F | TACTTCCAATCCAATGCCCGAGTTCTTGTCGTTGACGATGAC |  |
| ARR1-296-R | TTATCCACTTCCAATGTTATCCAAGCCGTCTTAGATATATCCGGTAT | For both pMCSG53 and pMCSG62 |
| pRSF-BirA-F | TACTTCCAATCCAATGCCATGAAGGATAACACCGTGCCACTGAAATTGA |  |
| pRSF-BirA-R | TTATCCACTTCCAATGTTATTTTTCTGCACTACGCAGGGATATTTCACC |  |
